# The complete mitochondrial genome of the *Pieris napi* (Lepidoptera: Pieridae) and its phylogenetic implication

**DOI:** 10.1080/23802359.2020.1797565

**Published:** 2020-07-28

**Authors:** Hong Yu, Min-Rui Shi, Jin Xu

**Affiliations:** Yunnan Academy of Biodiversity, Southwest Forestry University, Kunming, PR China

**Keywords:** *Pieris napi*, mitochondrial genome, phylogenetic analysis

## Abstract

In this study, the mitochondrial genome (mitogenome) sequence of *Pieris napi* is determined using next-generation sequencing. The entire mitogenome genome is determined to be 15,178 bp in length. It contains 37 genes, including 13 protein-coding genes (PCGs), 22 transfer RNA genes (tRNAs), two ribosomal RNA genes (rRNAs), and an adenine (A) + thymine (T)-rich region. The overall GC content of the genome is 19.9%. A phylogenetic tree reconstructed by 22 mitogenomes reveals that *P. napi* is most closely related to *Pieris melete*.

*Pieridae,* including about 1200 species are distributed worldwide and their hosts are Cruciferae, Leguminosae, Cauliflower, and Rosaceae (Chou 1998; Häuser et al. 2005). Most of its species are vegetable or fruit tree pests. They are generally medium in size, lighter in color, most are white or yellow, and a few are red or orange (Kemp et al. 2005). The Pieridae plays a very important role in the field of systematic evolution research. However, due to the lack of genomic information, there is still some controversy about the classification and phylogeny of these taxa. To date, there is less information about the complete mitochondrial genomes (mitogenomes) of *P. napi*. In our study, the complete mitogenome of *P. napi* is determined, which would provide basic genome information for further genetic studies, phylogenetic analysis, and conservation of this species.

Total genomic DNA was extracted from the thorax muscle of a single individual butterfly (N21°98′73.12″, E100°91′17.29″) using the Sangon Animal genome DNA Extraction Kit (Shanghai, China). The voucher specimen was deposited at Southwest Forestry University (AMCFD: 1808). The whole-genome sequencing was conducted by Hefei Biodata Biotechnologies Inc. (Hefei, China) on the Illumina Hiseq 4000 Sequencing System (Illumina, Hayward, CA). The filtered sequences were assembled using the SPAdes assembler version 3.10.0 (Bankevich et al. 2012). Annotation was performed using the BLAST+ (Camacho et al. 2009 ) and tRNAscan (Schattner et al. 2005).

The mitogenome (NCBI acc. no. MT576638) of *P. napi* was determined to comprise double stranded, circular DNA of 15,178 bp containing 13 protein-coding genes (PCGs), 22 transfer RNA genes (tRNAs), two ribosomal RNA genes (rRNAs), and an adenine (A) + thymine (T)-rich region with 91.5% AT content. Among these, 14 genes were encoded on the L-strand, including four PCGs (ND1, ND4, ND4Land ND5), two rRNA genes, eight tRNA genes (tRNAGlu, tRNACys, tRNATyr, tRNAPhe, tRNAHis, tRNAPro, tRNAleu, and tRNAVal) and A + T-rich region. The remaining 23 genes are encoded on the H strand. The arrangement of genes is similar to all know *Pieridae* species. The overall GC content of the genome is 19.9%.

To investigate its taxonomic status, a maximum-likelihood (ML) was reconstructed based on whole mitogenome from 20 *Pierinae* butterflies and two outgroup butterfly (*Argynnis hyperbiu*s and *Timelaea maculata*) by Mafft version 7.0 and FastTree version 2.1.10 (Price 2010; Katoh and Standley 2013). The ML phylogenetic tree shows that *P. napi* is most closely related to *Pieris melete*, with bootstrap support values of 100% (Figure 1).

**Figure 1.**
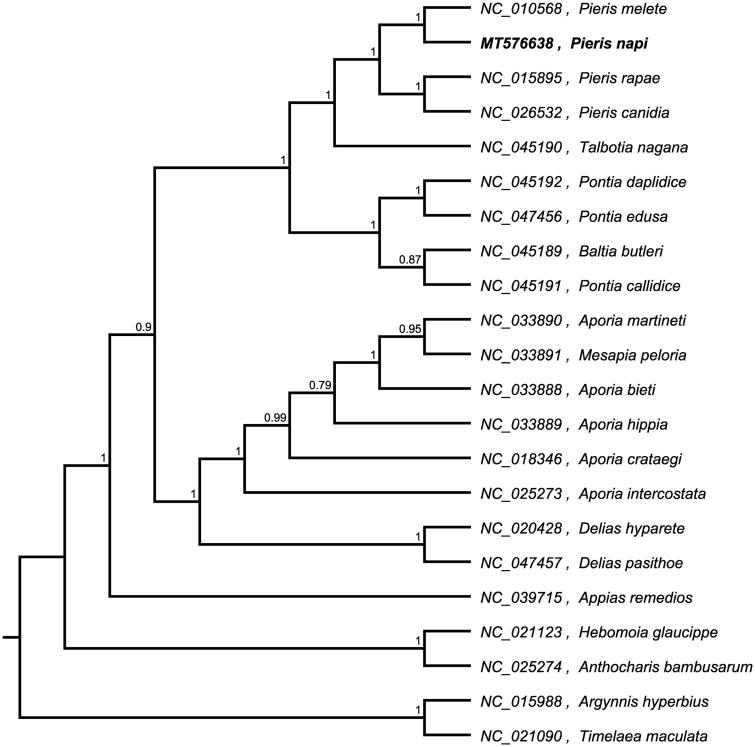
Maximum-likelihood phylogenetic tree based on whole mitogenome from 15 *Papilio* butterfly and two outgroup butterfly (*Argynnis hyperbiu*s and *Timelaea maculata*) and the support values are shown at the branches.

## Data Availability

The complete mitogenome sequence of *Pieris napi* is deposited in the GenBank database https://www.ncbi.nlm.nih.gov/ with the accession number MT576638.
